# Multi-atlas active contour segmentation method using template optimization algorithm

**DOI:** 10.1186/s12880-019-0340-6

**Published:** 2019-05-24

**Authors:** Monan Wang, Pengcheng Li, Fengjie Liu

**Affiliations:** School of Mechanical & Power Engineering, Harbin University of Science and Technology, Xue Fu Road No. 52, Nangang District, Harbin City, Heilongjiang Province 150080 People’s Republic of China

**Keywords:** Multi-atlas segmentation, Images registration, Active contour model, Template optimization

## Abstract

**Background:**

Brain image segmentation is the basis and key to brain disease diagnosis, treatment planning and tissue 3D reconstruction. The accuracy of segmentation directly affects the therapeutic effect. Manual segmentation of these images is time-consuming and subjective. Therefore, it is important to research semi-automatic and automatic image segmentation methods. In this paper, we propose a semi-automatic image segmentation method combined with a multi-atlas registration method and an active contour model (ACM).

**Method:**

We propose a multi-atlas active contour segmentation method using a template optimization algorithm. First, a multi-atlas registration method is used to obtain the prior shape information of the target tissue, and then a label fusion algorithm is used to generate the initial template. Second, a template optimization algorithm is used to reduce the multi-atlas registration errors and generate the initial active contour (IAC). Finally, a ACM is used to segment the target tissue.

**Results:**

The proposed method was applied to the challenging publicly available MR datasets IBSR and MRBrainS13. In the MRBrainS13 datasets, we obtained an average thalamus Dice similarity coefficient of 0.927 ± 0.014 and an average Hausdorff distance (HD) of 2.92 ± 0.53. In the IBSR datasets, we obtained a white matter (WM) average Dice similarity coefficient of 0.827 ± 0.04 and a gray gray matter (GM) average Dice similarity coefficient of 0.853 ± 0.03.

**Conclusion:**

In this paper, we propose a semi-automatic brain image segmentation method. The main contributions of this paper are as follows: 1) Our method uses a multi-atlas registration method based on affine transformation, which effectively reduces the multi-atlas registration time compared to the complex nonlinear registration method. The average registration time of each target image in the IBSR datasets is 255 s, and the average registration time of each target image in the MRBrainS13 datasets is 409 s. 2) We used a template optimization algorithm to improve registration error and generate a continuous IAC. 3) Finally, we used a ACM to segment the target tissue and obtain a smooth continuous target contour.

## Background

Brain image segmentation is crucial for the diagnosis and treatment of brain diseases, and it is the basis of the three-dimensional reconstruction of brain structure and quantitative analysis of lesions. The accuracy of segmentation directly affects focus tissue localization, lesion shape and size measurement, and clinical diagnosis and treatment planning.

Many brain diseases are manifested as changes in the normal volume and regional distribution of the WM, GM and cerebrospinal fluid (CSF), such as Alzheimer’s disease [1], which is characterized by atrophy of the whole brain, decrease in volume, narrowing of the brain at the cortex, and deepening of the cerebral sulci. Multiple sclerosis [2] shows ventricular enlargement and WM encroaching on GM. The thalamus is associated with epilepsy, and deep brain stimulation of thalamic nuclei is being developed as a treatment for drug- resistant epilepsy [3]. Reliably identifying the thalamus can increase the success rate of treatment. The thalamus belongs to the GM and is connected to not only the WM and CSF but also other structures belonging to the GM. This causes the boundaries of the thalamus to be inconspicuous and difficult to identify. Therefore, accurately identifying and analysing changes in the volume, shape, size, and grayscale distribution of these brain structures is very important for clinical diagnosis and treatment.

The image segmentation method based on the ACM [4] has a simple expression and high computational efficiency, and by this method, a smooth continuous target contour can be obtained. In recent decades, ACM has been successfully applied to image edge detection, image segmentation and motion tracking [5]. Chakraborty [6] integrated gradient and region information to achieve the target contour segmentation and showed that the integrated method performs better than conventional gradient-based image segmentation. However, ACM is very sensitive to the initial contour, and it is difficult to achieve accurate segmentation for target tissues with large intensity inhomogeneities and complex shapes. An effective way to solve this problem is to use prior knowledge of shape information to constrain contour deformation [7].

In recent years, multi-atlas segmentation (MAS) methods have become the basic tool for image segmentation. MAS methods can transfer the prior information of the atlas to the target image, and the segmentation accuracy is equivalent to that of manual segmentation [8, 9]. MAS methods use multi-atlas registration to select reference labels for the target image. The reference labels are manually created by the doctor according to the atlas images. Then, the transformation parameters obtained by multi-atlas registration are used to warp the reference labels. Finally, a label fusion method is used to correct the errors of each label. In recent years, many algorithms based on the MAS framework have been proposed, with particular emphasis on label fusion and atlas selection [10–12].

Artaechevarria [13] proposed a generalized local weighting voting (WV) method, which improved the segmentation accuracy in segmenting high-contrast structures compared to the global WV method. However, the contour obtained by the multi-atlas-based segmentation method is not smooth and is prone to breakpoints. Thomas et al. [14] proposed a selective and iterative method for performance level estimation (SIMPLE), which combines atlas selection and performance estimation strategies to achieve the segmentation of the target organization. Jimit et al. [15] proposed a multi-atlas region segmentation utilizing ensembles (MUSE), which improves segmentation accuracy by linear combinations of different atlas selections, deformation algorithms and deformation parameters. Zhang et al. [16] proposed a multi-atlas level set framework (MALSF) segmentation by combining the ACM and MAS method. This method uses multi-atlas registration to obtain the a priori information of the target tissue and then uses the level set algorithm to segment the target tissue and obtain a smooth, continuous contour.

Learning-based image segmentation methods have been shown to achieve satisfactory results when applied to image segmentation problems. Bai et al. [17] proposed a segmentation method combining augmented features and support vector machines (SVMs) and improved the performance of non-local patch-based segmentation. Zikic and Glocker [18] proposed a multi-atlas label propagation method based on randomized classification forests. Convolutional neural networks (CNNs) have shown superiority in several computer vision tasks, such as the ImageNet challenge [19]. Recently, CNNs have also been prevalent in medical image analysis [20] due to their flexibility. Moeskops et al. [21] presented a multi-scale patch-wise CNNs approach to segment brain images. It simplified tissue segmentation into a classification problem by extracting the patch in voxels and judging which tissue it belonged to. Subsequently, Nie et al. [22] employed fully convolutional networks (FCNs) to segment the isointense phase brain MR images. Hou et al. [23] proposed a 3D Convolutional Neural Network Segmentation Method Combined with Boundary Correction (BC-CNN). They used densely CNNs to roughly classify the imaged tissue and then detailed the boundaries generated in the previous stage.

In this paper, we propose a multi-atlas active contour segmentation method using a template optimization algorithm. The proposed method combines the advantages of a multi-atlas and ACM and can effectively utilize prior knowledge of the atlas and obtain a smooth target tissue contour. It is closer to reality and facilitates the three- dimensional reconstruction of the target tissue; thus, it is convenient for the doctor to observe the volume and morphological changes of the target tissue. To verify the segmentation performance of our method, we tested our method on the IBSR and MRBrainS13 datasets and employed four commonly used metrics, Dice, Recall, Precision and HD, to evaluate the similarity between segmentation results and manual labels. The results show that our method obtains satisfactory segmentation accuracy.

## Method

**Fig. 1:**
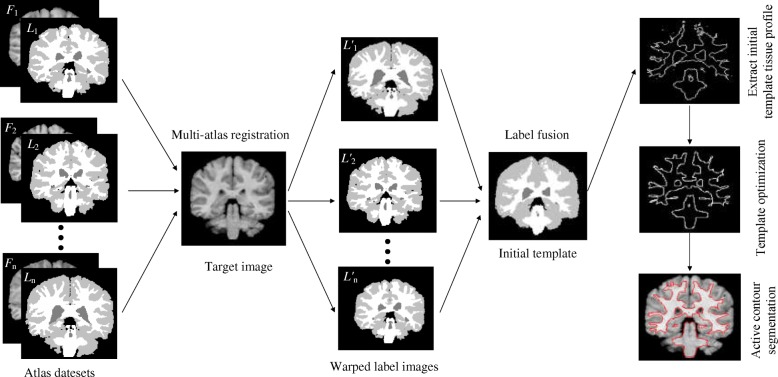
Schematic diagram of multi-atlas active contour segmentation method using template optimization algorithm

The overall framework of our method is summarized in Fig. 1. Our method has four steps: multi-atlas registration, label fusion, template optimization and ACM. Each step of our method is described in detail in the following subsections.

### Multi-atlas registration

An atlas contains an intensity image and a reference label that is manually divided by the doctors. In multi-atlas image registration, the intensity images are registered with the target image to obtain transformation parameters; then, the transformation parameters are used to warp the label images to spatially correspond to the target image. Finally, the warped label images are fused to generate the initial template. The multi-atlas-based segmentation method can effectively reduce the influence of individual atlas registration errors on segmentation results because a single atlas registration error may be rejected during label fusion.

In this paper, we use a global registration method based on affine transformation and then use a label fusion and template optimization algorithm to correct the errors caused by registration. The affine transform is defined as follows:

1 $$ {f}^{\prime}\left(x,y\right)=K\left[\begin{array}{cc}\cos \theta & -\sin \theta \\ {}\sin \theta & \cos \theta \end{array}\right]f\left(x,y\right)+\Delta X $$

Where *f*(*x*, *y*) is pixel coordinate, K is scaling parameter, θ is rotation angle and *Δx* is translation parameters. Considering the IBSR datasets from different magnetic imaging devices, there is non-standard intensity between images, therefore, we use normalized correlation coefficient (NCC) as the similarity measure function:

2 $$ NCC\left(\tau, TI, FI\right)=\frac{\sum_{i=1}^n\left( TI\left({x}_i\right)-\overline{TI}\right)\times {\sum}_{i=1}^n\left( FI\left(\tau \left({x}_i\right)\right)-\overline{FI\left(\tau \right)}\right)}{\sqrt{\sum_{i=1}^n{\left( TI\left({x}_i\right)-\overline{TI}\right)}^2\times {\sum}_{i=1}^n{\left( FI\left(\tau \left({x}_i\right)\right)-\overline{FI\left(\tau \right)}\right)}^2}} $$

3 $$ \overline{TI}={\sum}_{i=1}^n TI\left({x}_i\right)/n $$

4 $$ \overline{FI\left(\tau \right)}={\sum}_{i=1}^n FI\left(\tau \left({x}_i\right)\right)/n $$

Where *TI*(*x*_*i*_) is the gray value at pixel point *x*_*i*_ in the target image, *FI*(*x*_*i*_) is the gray value at pixel point *x*_*i*_ in the warped floating image and *n* represents the number of image pixels. The NCC assumes that there is a certain linear relationship between the two images, and it is widely used in single-modality medical image registration.

### Label fusion

After image registration, the target image pixels must be assigned a label. In multi-atlas registration, many atlas labels are generated; therefore, a label fusion algorithm is needed to fuse a plurality of warped label images to obtain a clear and accurate fusion image.

Label fusion is a key step in a multi-atlas-based registration segmentation method, and it can discard pixels with low agreement between different propagation labels to minimize outliers. Therefore, label fusion can effectively improve segmentation accuracy, but it may lead to slight distortions between the images.

In recent years, research on segmentation based on a multi-atlas has mainly focused on the label fusion method. Some label fusion methods have been proposed, such as majority voting [11], local WV [13], and probabilistic atlases [24], etc.

Voting rules are typically applied because they are computationally simple. Considering that there are large local differences in different atlas images, in this paper, we use a local WV algorithm to assign the appropriate label to the target image.

5 $$ S(x)=\arg \underset{c}{\max}\sum \limits_{i=1}^L{w}_i(x)\bullet f\left({M}_i^{\prime }(x),c\right) $$

Where c represents a class, *L* is the number of atlases, *w*_*i*_(*x*) is a weight function at pixel x, and each pixel is given a different weight according to the NCC similarity coefficient of the local area between atlas image and target image, in this paper, the local area size is set to 3 × 3. The $f\left({M}_i^{\prime }(x),c\right)$ is defined as:

6 $$ f\left({M}_i^{\prime }(x),c\right)=\left\{\begin{array}{cc}1& {M}_i^{\prime }(x)=c\\ {}0& {M}_i^{\prime }(x)\ne c\end{array}\right. $$

### Template optimization

The generated initial template is generally similar to the target label image; however, there is still a large local error. In our method, we use a template optimization algorithm to correct registration errors. It mainly consists of three steps: search area setting, contour point determination, and IAC acquisition.

#### Search area setting

There are three steps in the search area setting:

1. Extracting the WM contour of the initial template
2. Selecting a certain size rectangular area as the initial search area centred on each pixel on the contour
3. Determining whether each pixel in the search area belongs to the contour boundary according to the grayscale and gradient information of the target image

**Fig. 2:**
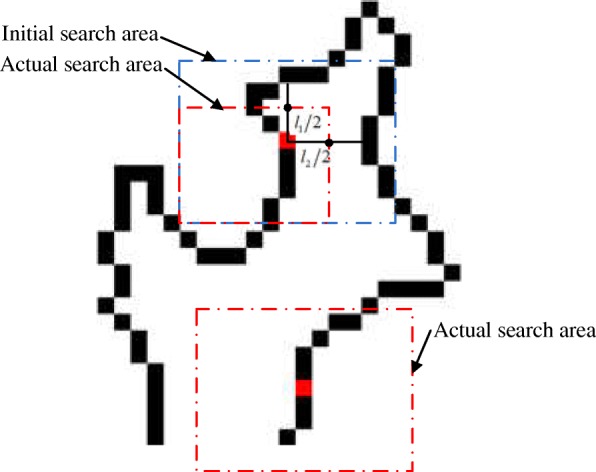
Search area setting: black curve is tissue outline, red pixel is center pixel, blue dotted line is initial search area, and the red dotted line is the actual search area

To avoid search area overlap problems from two adjacent outlines, we adjust the scope of the search area. As shown in Fig. 2, if there is an outline that is not adjacent to the central pixel in the x-direction or y-direction in the initial searched area, the search area boundary value is set to the middle value between the two contour lines. The adjusted search area is then defined as the actual search area.

#### Contour point determination

**Fig. 3:**
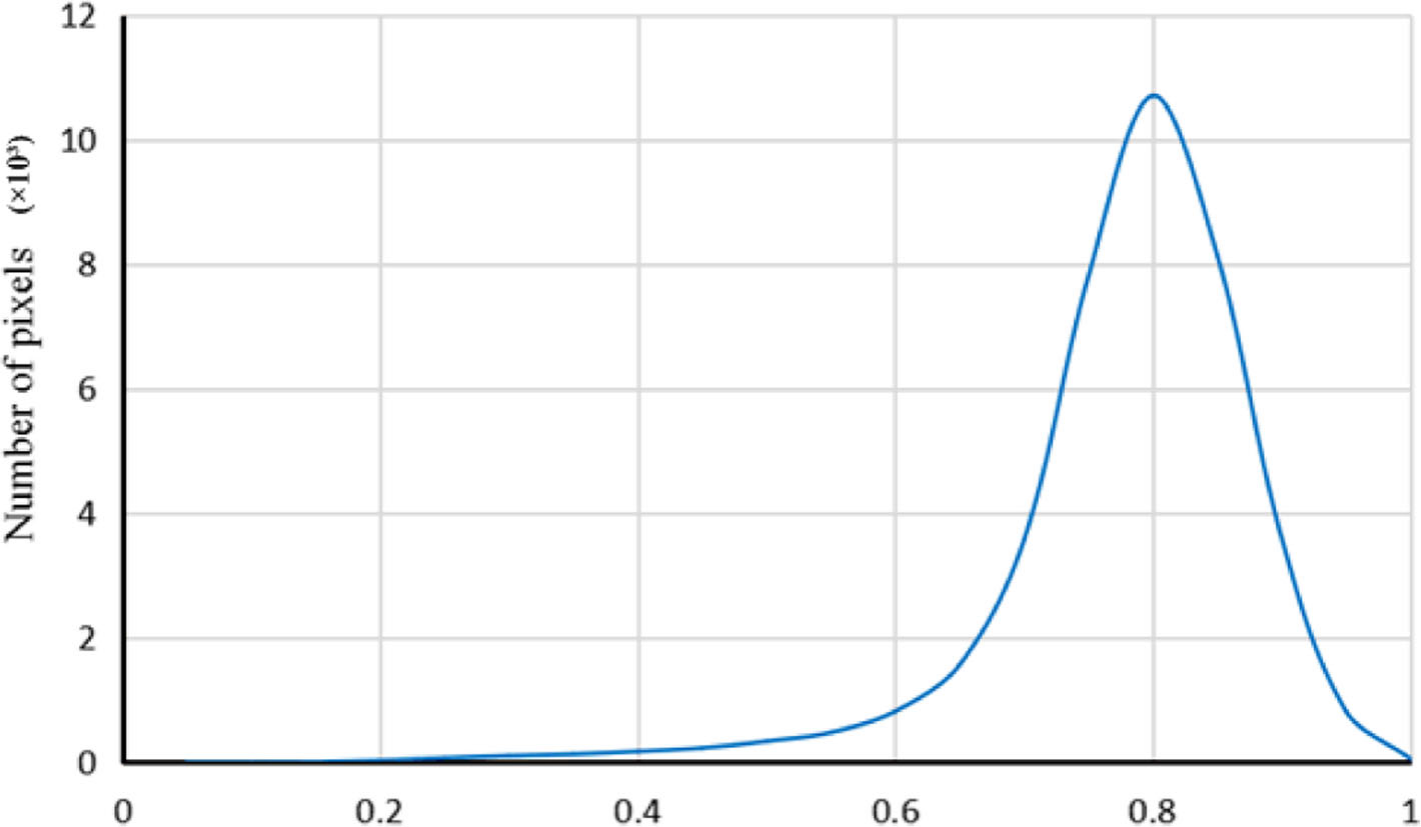
Intensity distribution map of the pixels on the WM contour of the IBSR datasets

To avoid the effect of noise, we use a Gaussian kernel function with a standard deviation of 0.5 to smooth the target image and the grayscale images in the IBSR datasets. We also perform pixel value normalization on these images. Because the boundary gradient between WM and GM is small, it is not ideal to determine whether a pixel is a contour point based on the local gradient value. Therefore, we introduce the gray information of the target image. We extract the intensity distribution map of the pixels on the WM contour of the IBSR datasets. As shown in Fig. 3, the intensity distribution map approximates a normal distribution. The pixel value from 0 to 1 is evenly divided into 20 intervals, and an intensity weight is assigned for each pixel according to the intensity distribution map. The weight value is defined as follows:

7 $$ P(x)=\frac{pr(x)-\min pr}{\max pr-\min pr} $$

8 $$ pr(x)={n}_i/\sum \limits_{i=\mathrm{l}}^N{n}_{i\kern6.5em i=1,2\cdots 20} $$

Where *i* is the pixel value interval number corresponding to the pixel *x*, *n*_*i*_ is the number of pixel points in the interval *i*.

Because the MR image’s intensity is not constant for each tissue type, this results in a large gradient change at the WM profile. It is difficult to find a suitable gradient threshold to identify the contour boundary; to address this problem, we use the Robert edge detection operator [25] to calculate the gradient of each pixel in the actual search area and normalize the gradient. Then, we combine the intensity weight and gradient value to determine if the pixel is a contour point. The decision formula is defined as follows:

9 $$ Y={w}_p\bullet P(x)+{w}_g\bullet \frac{\mathit{\operatorname{grad}}(x)-\min \mathit{\operatorname{grad}}}{\max \mathit{\operatorname{grad}}-\min \mathit{\operatorname{grad}}} $$

where grad() is the pixel gradient and are weight coefficients. The range of Y is from 0 to 1. The larger the Y value, the greater the probability that the pixel point is the contour point. The tissue contour within the actual search area can be extracted by setting a suitable threshold for Y.

#### IAC acquisition

In this paper, we use a parameter ACM to segment the target tissue. The parameter ACM needs to set a continuous IAC curve. However, the contour points obtained by the above method are not continuous, and there is no topological relationship between each point. To solve this problem, we use the contour points obtained in the above method to generate a tissue contour image and then add the topological relationship for each point according to the tissue contour image to obtain the IAC.

### Active contour model

In order to obtain smooth and accurate segmentation results and effectively use the prior information of the atlas images and the gray information of the target image, we use the parametric ACM to further correct the errors caused by the multi-atlas registration process. The energy equation is as follows:

10 $$ {E}_{snake}\left(v(s)\right)=\underset{0}{\overset{1}{\int }}\left({E}_{\mathrm{int}}\left(v(s)\right)+{E}_{ext}\left(v(s)\right)\right) ds $$

The ACM is actually a functional of a plane curve, and the target contour curve is the local minimum of the functional. Where *E*_*int*_ is the internal energy term and is defined as follows.

11 $$ {E}_{\mathrm{int}}\left(v(s)\right)=\frac{1}{2}\int \alpha (s){\left|{v}^{\prime }(s)\right|}^2+\beta (s)\left|{v}^{{\prime\prime} }{(s)}^2\right| ds $$

Where *v*^′^(*s*) is the first order derivative of the IAC, it represents the elastic energy of the curve. The *v*^″^(*s*) is the second order derivative of the IAC, it represents the bending energy of the curve. *α*(*s*) , *β*(*s*) are weight parameters, *α*(*s*) controls the continuity of the IAC and *β*(*s*) controls the smoothness of the IAC.

*E*_*ext*_ is the external energy term, and it is used to pull the IAC toward target image edges. The definition of the *E*_*ext*_ is the key to the ACM. In our method, the *E*_*ext*_ is defined as follows:

12 $$ {E}_{ext}\left(v(s)\right)=\underset{\Omega}{\int}\gamma (s){E}_{img}\left(v(s)\right) ds $$

where *E*_*img*_ is a scalar potential function defined on the image plane [26], *γ*(*s*) is weight parameter of *E*_*img*_*.* Generally, the gradient of the target image is used to build the *E*_*img*_. But there is a restriction that the *E*_*img*_ sometimes will converge the target contour to the wrong border, such as the border between the background and the brain tissue. To avoid this problem, we combine image grayscale and gradient information to build the *E*_*img*_:

13 $$ {E}_{img}(x)=\left|\nabla {\left({G}_{\sigma}\ast I(x)\right)}^2+P(x)\right| $$

where *I*(*x*) is the initial grayscale image, *G*_*σ*_ is a Gaussian smoothing filter. The ∇ symbol represents the gradient operator.

## Results

In this section, we tested our method on the ground- truthed IBSR datasets and MRBrainS13 datasets. The image datasets and the performance of each step of our method are described in detail in the following subsections.

### Data and segmentation evaluation index

We tested our method using multi-center image data with a total of 55 image datasets from multiple scanners in the IBSR datasets [27] and MRBrainS13 datasets. Multi-center image data are superior to a single center in that the former contain a large amount of image data from different regions, different people, and different scanners. These factors will make the image intensity and structure different. Therefore, the application of multi-center image data can better evaluate the applicability and segmentation performance of different segmentation methods.

The IBSR datasets contain 38 normal T1-weighted MR brain datasets and their reference regions of interest (ROI) labels, which are defined by the Center for Morphometric Analysis at Massachusetts General Hospital, and all the data were publicly available. Twenty datasets are low-resolution data whose dimensions are 256 × 256 × 60, and the remaining eighteen datasets are high-resolution data whose dimensions are 256 × 256 × 128. In the low-resolution datasets, organizational boundaries are more difficult to identify. In our method, a template optimization algorithm is used to identify this boundary information. To verify the validity of our method, we tested it in the 20 low-resolution datasets.

MRBrainS13 datasets were provided by https://masi.vuse.vanderbilt.edu/. This website provides the online continuation of a segmentation contest held at the 2013 Medical Image Computing and Computer Assisted Intervention Challenge (MICCAI). There are 35 T1-weighted MR brain image datasets and their label images. The dimensions of all datasets are 256 × 256 × 287.

We employed three commonly used metrics, Dice, Recall and Precision, to measure the volumetric overlap of the segmentation results with manual labels, and we used HD to measure the surface distance between segmentation results and manual labels. These metrics are defined as follows:

$$ Dice\left(T,F\right)=\frac{2V\left(T\cap F\right)}{V(T)+V(F)} $$ $$ \operatorname{Re} call\left(T,F\right)=\frac{V\left(T\cap F\right)}{V(T)} $$ $$ \Pr ecision\left(T,F\right)=\frac{V\left(T,F\right)}{V(F)} $$ (14)

15 $$ HD\left(T,F\right)=\max \left({H}_1\left(T,F\right),{H}_2\left(F,T\right)\right) $$

16 $$ {H}_1\left(T,F\right)=\underset{p_t\in T}{\max}\left(\underset{p_f\in F}{\min }d\left({p}_t,{p}_f\right)\right) $$

17 $$ {H}_2\left(F,T\right)=\underset{p_f\in F}{\max}\left(\underset{p_t\in T}{\min }d\left({p}_f,{p}_t\right)\right) $$

Where *T* is the target tissue pixel set in the target label image, and *F* is the segmented target tissue pixel set. *HD*(*T*, *F*) is the HD between the pixel set *T* and *F*, *d*(*p*_*t*_, *p*_*f*_) is the distance between pixel *p*_*t*_ and *p*_*f*_.

We used a leave-one-out cross-validation method to test the performance of our method on the IBSR and MRBrainS13 datasets. One MR brain dataset was selected as the target set, and the remaining MR brain datasets were treated as training sets; this was repeated until each MR brain dataset was used as a target set.

### Label fusion

To reduce the influence of background pixels on the registration result, we took the smallest rectangular area that contains all the target image brain tissues as the ROI. In this step, we selected the label images with NCC values greater than 0.87 after image registration as the label images corresponding to the target image. We then used local WV to fuse these label images to generate the initial template with the binarization threshold set to 0.5.

**Fig. 4:**
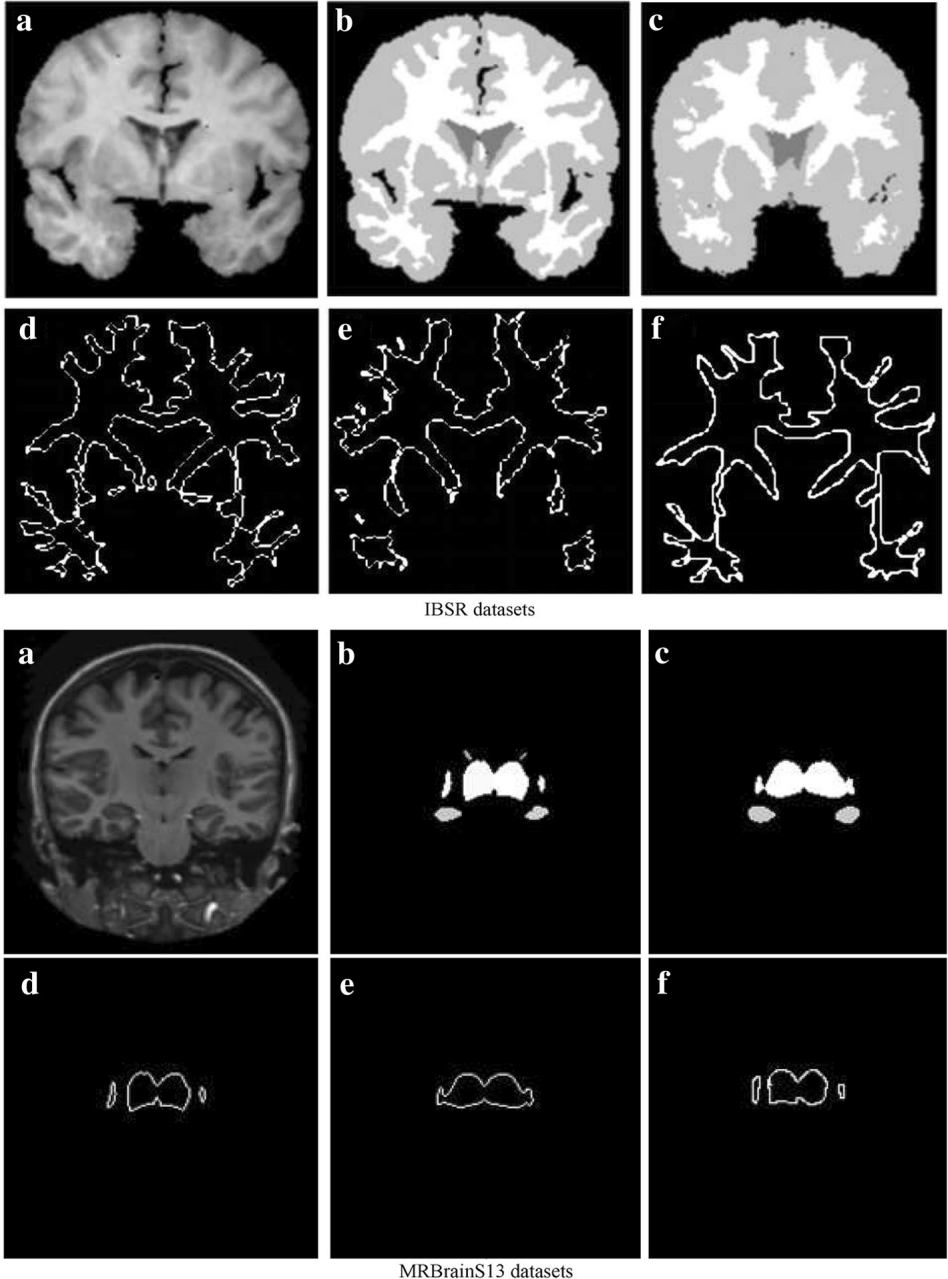
Label fusion and Template optimization: **a** target image. **b** Target label image. **c** initial template. **d** Target tissue contour in label image. **e** Target tissue contour in initial template. **f** generated IAC

Figure 4 shows the results obtained during label fusion and template optimization. It can be seen from (c) and (b) that the generated initial template is generally similar to the target label image, but some small WM tissues in the brain returned are lost in the IBSR datasets, and some GM tissues are misdivided into the thalamus in the MRBrainS13 datasets. To reduce these errors, we used a template optimization algorithm.

### Template optimization

**Fig. 5:**
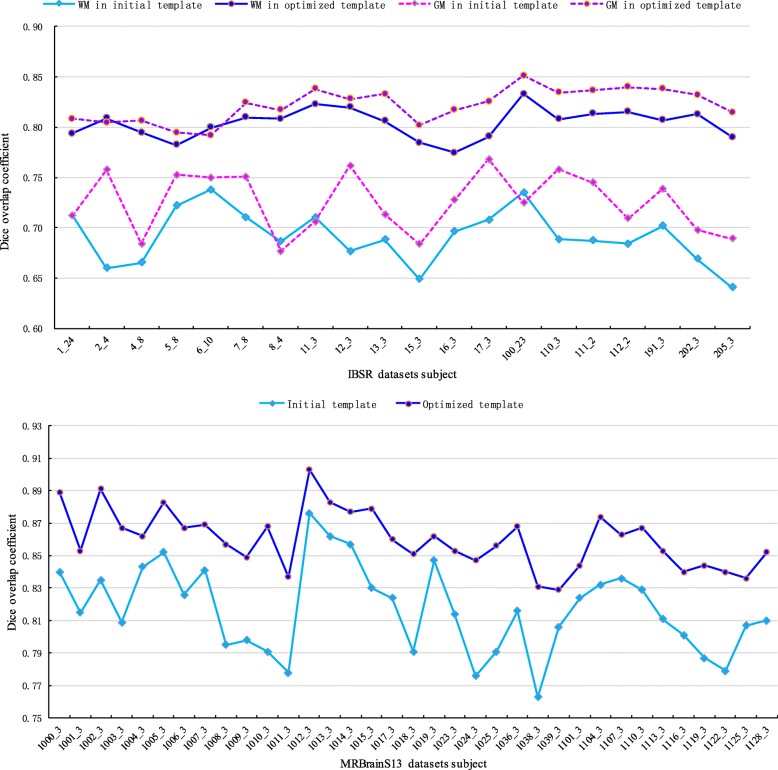
Dice similarity coefficients of the initial template and the optimized template

In this step, we used the template optimization algorithm to optimize the initial template. The optimization parameters are shown in Table 1. In Fig. 4, the (d), (e), and (f) images show the changes in the target tissue contour during the template optimization process. (f) is the template-optimized IAC; compared to the contour in (e), (f) adds some small target tissue contours and removes some of the wrong incorrect contour points. The IAC is a smooth continuous contour for the initial contour setting of the next step. Figure 5 shows the Dice similarity coefficients of the initial template and the optimized template. After template optimization, the Dice similarity coefficients were significantly improved.

**Table 1:** The parameters used in all experiments

Parameter		Template optimization				Active contour model		
		initial search area	*w*_*p*_	*w*_*g*_	*Y*	*α*(*s*)	*β*(*s*)	*γ*(*s*)
Value	IBSR	8	0.7	0.3	0.6	30	70	70
	MRBrainS13	8	0.6	0.4	0.6	60	100	100

### Experimental results

A parameter ACM was used to drive the IAC deformation to segment the target tissue, and the parameter settings are shown in Table 1. Table 2 shows the performance of the proposed method in the image segmentation of each subject. In the IBSR datasets, we obtained an average WM Dice coefficient of 0.853 ± 0.03 and an average WM Dice coefficient of 0.827 ± 0.04. Subject 100_23 had the best segmentation performance, with a WM Dice coefficient of 0.897 and a WM Dice coefficient of 0.873, while subject 5_8 had the lowest GM Dice coefficient of 0.806, and subject 15_3 had the lowest WM Dice coefficient of 0.773. In the thalamic segmentation of the MRBrainS13 dataset, we obtained an average Dice coefficient of 0.927 ± 0.014 and an average HD of 2.92 ± 0.53. Subject 1019_3 had the best segmentation performance, with a Dice coefficient of 0.957 and an HD of 1.414. Subject 1002_3 had the worst segmentation performance, with a Dice coefficient of 0.896 and an HD of 4.472.

**Table 2:** Performance index of our method for IBSR datasets and MRBrainS13 datasets. D_GM_,D_WM_ and D_Th_ represent the average dice similarity coefficient of GM, WM and thalamics. D_Th_ represents the average HD of thalamics

IBSR datasets:				Average D_GM_		0.853	Average D_WM_			0.827	
subject	D_GM_	D_WM_	subject	D_GM_	D_WM_	subject	D_GM_	D_WM_	subject	D_GM_	D_WM_
1_24	0.822	0.803	2_4	0.829	0.790	4_8	0.809	0.803	5_8	0.806	0.797
6_10	0.812	0.793	7_8	0.832	0.839	8_4	0.834	0.817	11_3	0.889	0.861
12_3	0.823	0.855	13_3	0.877	0.860	15_3	0.819	0.773	16_3	0.869	0.814
17_3	0.893	0.822	100_23	0.897	0.873	110_3	0.883	0.810	111_2	0.875	0.854
112_2	0.878	0.865	191_3	0.866	0.848	202_3	0.887	0.833	205_3	0.855	0.836
MRBrainS13 datasets:				Average D_Th_		0.927	Average H_Th_			2.923	
subject	D_Th_	H_Th_	subject	D_Th_	H_Th_	subject	D_Th_	H_Th_	subject	D_Th_	H_Th_
1000_3	0.928	2.828	1001_3	0.896	4.472	1002_3	0.914	3.162	1003_3	0.934	2.236
1004_3	0.92	2.828	1005_3	0.921	3.162	1006_3	0.92	3.606	1007_3	0.938	2.236
1008_3	0.942	2.828	1009_3	0.928	2.828	1010_3	0.916	3.162	1011_3	0.924	2.828
1012_3	0.903	4.123	1013_3	0.924	3.162	1014_3	0.941	2.236	1015_3	0.937	2.828
1017_3	0.91	3.606	1018_3	0.937	2.828	1019_3	0.957	1.414	1023_3	0.917	3.606
1024_3	0.93	2.828	1025_3	0.931	2.282	1036_3	0.94	2.236	1038_3	0.931	2.828
1039_3	0.906	3.606	1101_3	0.943	2.236	1104_3	0.936	2.828	1107_3	0.948	2.236
1110_3	0.932	2.828	1113_3	0.937	2.828	1116_3	0.918	3.606	1119_3	0.915	3.162
1122_3	0.921	3.162	1125_3	0.907	2.828	1128_3	0.938	2.828			

**Fig. 6:**
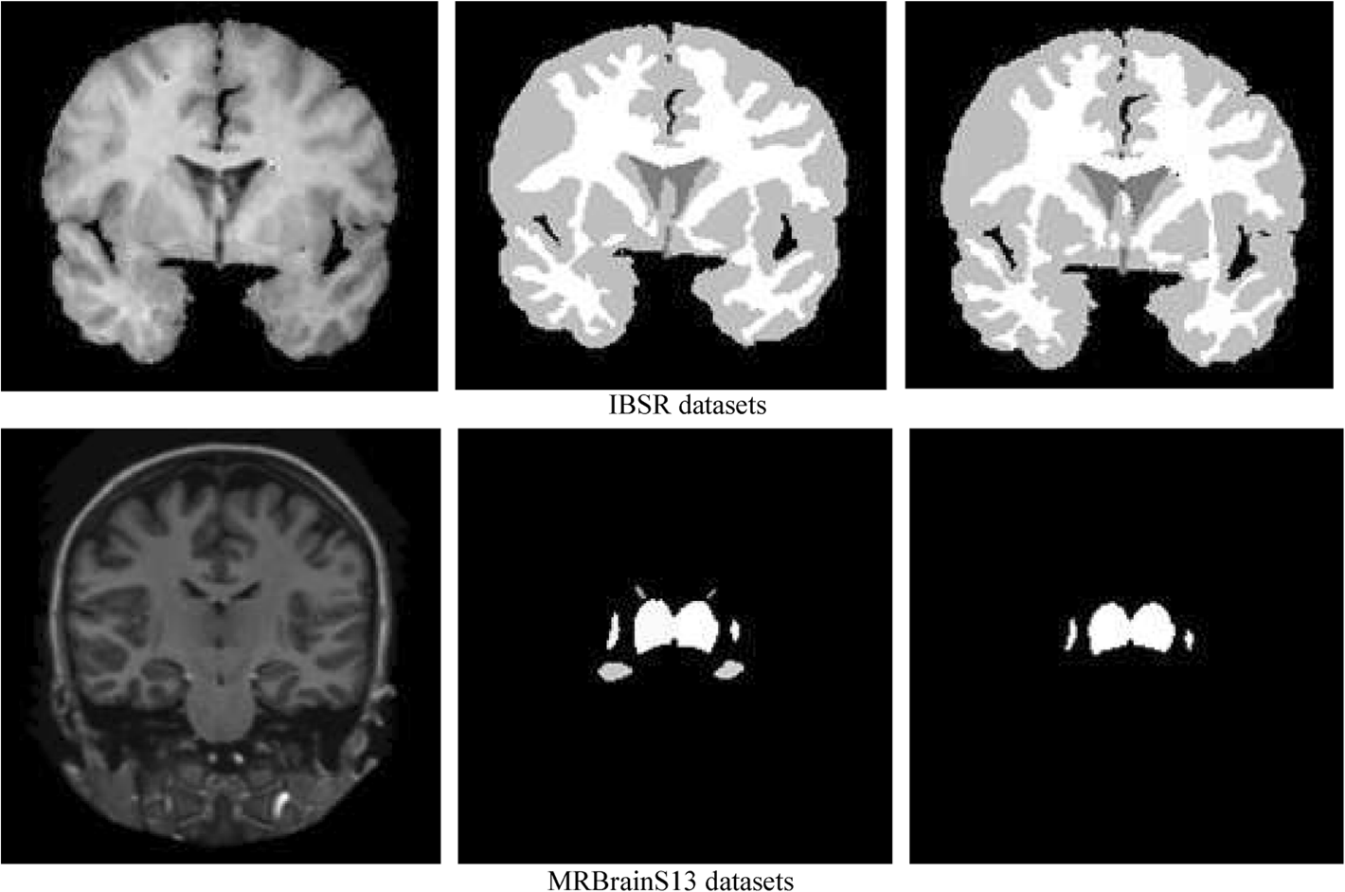
Sample segmentation result: Left: target image. Middle: target label image. Right: segmentation result

A sample of our segmentation results is shown in Fig. 6. It can be observed that our method obtained satisfactory WM, GM and thalamic segmentation performances, and the target organization boundary is smooth, which is beneficial to the three-dimensional reconstruction of the target organization.

**Fig. 7:**
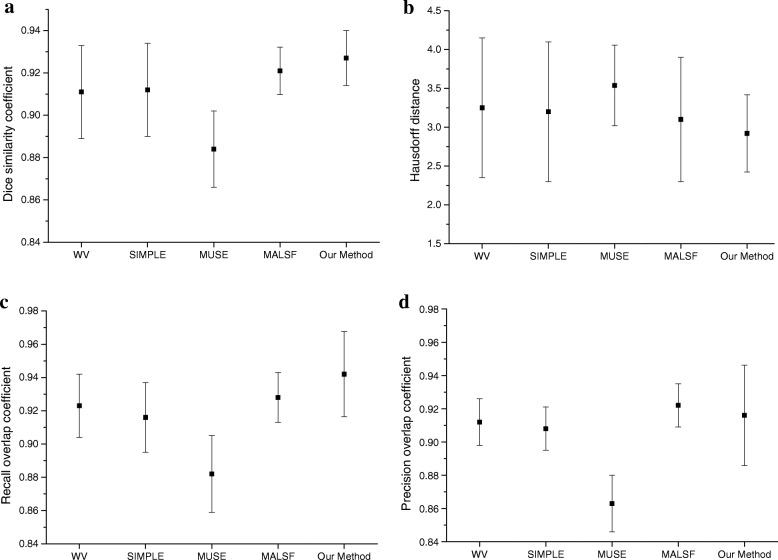
Performance indexes for the thalamic segmentation of the MRBrainS13 datasets, (**a**) Dice similarity coefficient, (**b**) Hausdorff distance, (**c**) Recall overlap coefficient, (**d**) Precision overlap coefficient

Figure 7 shows the means and standard deviations of the Dice, Recall, Precision and HD coefficients in the MRBrainS13 datasets. It can be observed from Fig. 7a and Fig. 7b that the proposed method results in a substantially higher average similarity coefficient and a smaller HD than WV [13], SIMPLE [14], MUSE [15] and MALSF [16]. The target organization segmented by our method is closer to manual expert segmentation than the methods mentioned above. As shown in Fig. 7c and Fig. 7d, the proposed method obtains the best Recall coefficient and the second-best Precision coefficient. This means that the proposed method results in the fewest unrecognized target tissue pixel points, while MALSF results in the fewest misidentified target tissue pixel points.

Currently, our method is implemented with Visual Studio 2012 on an Intel(R) Core(TM) 3.70 GHz CPU, and the average time for segmenting a new image was approximately 2 min.

## Discussion

Usually, MR image analysis is performed by a doctor through the identification and marking of various tissues in the image. However, this work requires a greater professional level of doctor and has high subjectivity. Automatic and semi-automatic image segmentation methods can effectively remove human subjective influences and can effectively reduce segmentation time and improve segmentation accuracy. This kind of method has become a research hotspot in the field of image segmentation.

At present, many automatic and semi-automatic segmentation methods have been proposed. Valverde et al. [28] tested several commonly used automatic image segmentation methods on IBSR datasets, such as fuzzy c-means (FCM) [29] and SPM8 [30]. Tohka et al. [31] proposed a novel MR image segmentation method (SVPASEG) based on a local Markov random field (MRF) and obtained satisfactory brain MR image segmentation results. Mahmood et al. [32] proposed an automatic MR image segmentation method combining mean shift, a priori spatial tissue probability maps and FCM (PPM- FCM). Bendib et al. [33] extended the stationary wavelet transform feature extraction method and used the extracted features to feed a random forest classifier (SWT- RF). They trained and tested this classifier on the IBSR datasets and obtained better WM and GM segmentation results.

**Fig. 8:**
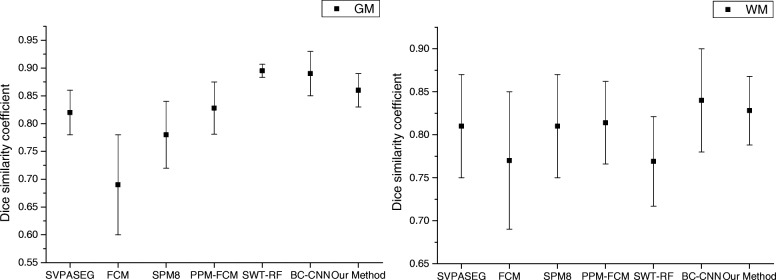
Average dice similarity coefficient for our method and other segmentation methods on the IBSR 20 normal brain scans

Figure 8 shows the performance of our method and the above methods on the IBSR datasets. In WM segmentation, the proposed method obtained the second highest Dice coefficient, similar to that of the BS-CNN method. In GM segmentation, the BC-CNN method obtained the best segmentation performance, and we obtained the third highest Dice coefficient. This is mainly due to the errors generated in the WM segmentation process affecting the performance of the GM segmentation. To verify the effect of WM segmentation errors on GM segmentation, we used subject 205_3 as the target dataset and introduced the WM segmentation errors into the target label image. We obtained an average GM Dice coefficient of 0.887 between the original target label image and the images with errors. This shows that the best Dice similarity coefficient for GM segmentation is 0.887 due to the effect of WM segmentation errors, and we obtained a GM segmentation result of 0.855.

Multi-atlas registration results have been shown to have a greater impact on segmentation accuracy [16]. The registration result is mainly affected by two factors. One is the choice of registration method, and the other is the selection of the atlas datasets. The more similar the target image and the images of the atlas dataset are, the better the multi-atlas registration results that will be obtained. Selecting a high-precision nonlinear registration method can achieve better registration results, but these methods are expensive to calculate. In the multi-atlas registration, nearly one thousand image registration steps would be carried out, and it would thus take a long time to use the highly complex nonlinear registration method. To reduce the multi-atlas registration time, we chose an image registration method based on affine transformation and then used the template optimization algorithm to improve the registration-induced errors. In our test, the multi-atlas registration process for each target image in the IBSR datasets took approximately 255 s, and in the MRBrainS13 datasets took approximately 409 s.

In the IBSR datasets WM segmentation, we obtained an average Recall coefficient of 0.836 ± 0.028 and an average Precision coefficient of 0.823 ± 0.034. In the IBSR datasets GM segmentation, we obtained an average Recall coefficient of 0.860 ± 0.037 and an average Precision coefficient of 0.849 ± 0.018. The Recall coefficients are higher than the Precision coefficients in the segmentation of the two datasets, which means that the pixel points misidentified as the target tissue in the segmentation result are relatively large. In the next study, we will analyse the reasons for this result.

Although the proposed method has been successfully applied to both IBSR datasets and MRBRainS13 datasets, there are still some limitations that affect segmentation accuracy and speed, and these limitations can be further improved. The proposed method can also be used for segmentation of different tissues of other datasets. In our next work, we will improve the limitations of the proposed method and apply it to the segmentation of different organizations of more datasets.

### Limitations

1. The initial template quality has a great impact on the segmentation results and is affected by the selection of atlas datasets and registration methods.
2. In our experiments, the initial search area was set according to the organization outline of the initial template during the template optimization process. If there is a large difference between the images, the template optimization method may not find the real boundary. For example, the location and shape of the diseased tissue have a large randomness, which results in a large difference between the images.
3. The multi-atlas registration process takes a long time, but not all image registrations are meaningful, such as registration between two very different images.
4. The proposed method cannot adapt to the segmentation of small-thickness tissues (such as cortex), because the target profile with small thickness will cause ACM to fall into local extremes.

### Future directions

1. Combine different atlas datasets and different registration methods to improve initial template quality.
2. Expand the initial search area and redefine the appropriate weight function to expand the capture range of the tissue contour points.
3. Select appropriate images in the atlas datasets for multi-atlas registration will not only save much time but also not reduce the accuracy of the segmentation. How to go about choosing the appropriate images will be a challenging exercise.

## Conclusion

In this paper, we propose a semi-automatic brain image segmentation method. First, a multi-atlas registration method is used to obtain the prior shape information of the target tissue, and then a label fusion algorithm is used to generate the initial template. Second, a template optimization algorithm is used to reduce the multi-atlas registration errors and generate a IAC. Finally, a ACM is used to segment the target tissue. Our method combines the advantages of multi-atlas registration and ACM. The IAC of the target tissue is obtained by multi-atlas registration and template optimization, and it can effectively reduce the influence of human subjective factors compared to manually setting the IAC. Then, a ACM is used to obtain a smooth, continuous target contour. (Fig. 4).

To verify the performance of our method in brain segmentation, we validated our method on two datasets, the IBSR and MRBrainS13 datasets. Experimental results demonstrate the following:

1) Compared with [16], we use the multi-atlas registration method based on affine transformation and use template optimization to improve the registration error. Our method effectively reduces the multi-atlas registration time and achieves higher segmentation accuracy.

2) Compared with the currently used automatic segmentation method, our method obtains better segmentation performance.

## Abbreviations

ACM: Active contour model
BC-CNN: 3D Convolutional Neural Network Segmentation Method Combined with Boundary Correction
CNNs: Convolutional neural networks
CSF: Cerebrospinal fluid
FCM: Fuzzy c-means
FCNs: Fully convolutional networks
GM: Gray matter
HD: Hausdorff distance
IAC: Initial active contour
MALSF: Multi-atlases level set framework
MAS: Multi-atlas segmentation
MRF: Markov random field
MUSE: Multi-atlas region segmentation utilizing ensembles
NCC: Normalized correlation coefficient
PPM-FCM: An automatic MR image segmentation method combining mean shift, priori spatial tissue probability maps and FCM
ROI: Regions of interest
SIMPLE: A selective and iterative method for performance level estimation
SVM: Support vector machine
SVPASEG: A MR image segmentation method
SWT-RF: A segmentation method combining stationary wavelet transform and random forest
WM: White matter
WV: Weighting voting
